# Overexpression of *cryIAb* gene for enhancing yellow stem borer (*Scirpophaga incertulas*) resistance using a marker-free double T-DNA system in Rojolele of Indonesia Rice cultivar

**DOI:** 10.1016/j.jgeb.2026.100782

**Published:** 2026-08-10

**Authors:** Agus Rachmat, Enny Rimita Sembiring, Ade Nena Nurhasanah, Nurul Fitriah, Vincentia Esti Windiastri, Eva Erdayani, Rumella Simarmata, Inez H.S. Loedin, Sri Indrayani, Carla Frieda Pantouw, Siti Halimah Larengkeng, Enung Sri Mulyaningsih

**Affiliations:** a Research Center for Genetic Engineering, National Research and Innovation Agency, Indonesia; b Research Center for Applied Botany, National Research and Innovation Agency, Indonesia; c Research Center for Applied Microbiology, National Research and Innovation Agency, Jl. Raya Jakarta-Bogor Km.46, Cibinong, Bogor, Jawa Barat 16911, Indonesia; d International Rice Research Institute, Los Baños, Philippines; e Faculty of Forestry, Hasanuddin University, Makassar, Indonesia

**Keywords:** *Oryza sativa*, Marker-free transformation, Double T-DNA, *cry1Ab*, *Scirpophaga incertulas*, Pest resistance

## Abstract

The aromatic rice cultivar Rojolele (*Oryza sativa* L. cv. Rojolele), prized for its superior organoleptic qualities, is highly susceptible to yellow stem borer (*Scirpophaga incertulas*), a pest causing significant yield losses in Asia. To address this vulnerability, we developed marker-free transgenic Rojolele lines expressing the *cry1Ab* gene using a double T-DNA vector system, eliminating reliance on antibiotic resistance markers. *Agrobacterium*-mediated transformation yielded 21 independent T_0_ lines, with PCR and Southern blot analysis confirming stable integration of *cry1Ab* in seven lines by the T_3_ generation. Immunostrip assays verified functional Cry1Ab protein expression, while bioassays demonstrated complete resistance in lines I.AR.4 and III.AR.5.3. Susceptible lines (I.AR.2, III.AR.5.2) exhibited variable transgene expressions, highlighting the importance of event selection. The marker-free strategy aligns with biosafety regulations, and the retained agronomic traits ensure compatibility with commercial cultivation. This study provides a sustainable solution to protect high-value aromatic rice, combining insect resistance with consumer-preferred qualities. Field trials and resistance management strategies, such as gene pyramiding, are recommended for future deployment.

## 1 Introduction

Rice (*Oryza sativa* L.) serves as the primary caloric source for more than half of the world's population, yet its productivity faces significant challenges from insect pests, particularly the monophagous yellow stem borer (*Scirpophaga incertulas* Walker). This lepidopteran pest, whose geographical distribution correlates strongly with precipitation during the wettest month and mean annual temperature,^1^ induces yield losses ranging from 10% to 90% through deadheart and whitehead formation^1^, ^2^ establishing it as Asia's most devastating rice pest, including in Indonesian agroecosystems.^3^

The soil bacterium *Bacillus thuringiensis* (Bt) has emerged as a pivotal biological control agent, producing crystalline (Cry) proteins with selective toxicity against Coleoptera, Diptera, and Lepidoptera^4^, ^5^ These parasporal inclusions, when ingested by target insects, undergo proteolytic activation in the alkaline midgut, subsequently binding on specific receptors (e.g., cadherins and aminopeptidases). This triggers a cascade involving G-protein signaling, adenylate cyclase activation, and PKA-mediated cell lysis, ultimately causing insect mortality^6^˒.^7^

Current reliance on chemical pesticides for *S. incertulas* management presents dual risks of environmental contamination and rapid resistance evolution.^8^ Conventional breeding approaches remain constrained by limited resistant germplasm and complex inheritance patterns. Bt toxins (e.g., Cry proteins) provide a sustainable solution by selectively targeting insect midgut receptors while remaining safe for non-target organisms^7^˒^5^˒.^2^ Consequently, transgenic strategies incorporating Bt toxins (e.g., Cry1Ab, Cry1C, Cry2A) have demonstrated remarkable success in japonica and indica cultivars, reducing pesticide dependence while maintaining ecological safety^9^˒.^10^ This approach is exemplified by the recent development of marker-free transgenic *Bt* rice expressing a novel chimeric *cry2AX1* gene, which confers resistance to both the yellow stem borer and leaf folder while addressing key biosafety and regulatory concerns.^11^ However, the long-term success of Bt crops depends on stable toxin expression and resistance management,^12^ and emerging challenges include pest resistance evolution linked to gut microbiome dynamics.^13^ Recent advances, such as gene pyramiding^14^˒,^15^ and the development of marker-free systems as exemplified by^11^, are critical to address these challenges and regulatory concerns.^16^

Notably, most commercialized Bt rice varieties lack aromatic qualities, creating an unmet demand for resistant, premium fragrance cultivars. Rojolele, a tropical japonica landrace from Klaten, Indonesia, represents a high-value aromatic rice characterized by exceptional organoleptic properties but susceptibility to stem borers.^17^ Enhancing this cultivar through targeted integration of *cry1Ab* via advanced marker-free transformation could simultaneously address pest vulnerability and market preferences.

This study implements a dual T-DNA vector system to generate marker-free Rojolele lines featuring stable transgene integration (confirmed by PCR and Southern blot), complete marker excision by the T₃ generation, and strong resistance to *Scirpophaga incertulas* in bioassays, thereby bridging the gap between insect resistance and culinary quality. Our work establishes a framework for improving specialty crops through precision biotechnology.

## 2 Materials and methods

### 2.1 Vector construction containing double T-DNA

**Fig. 1:**
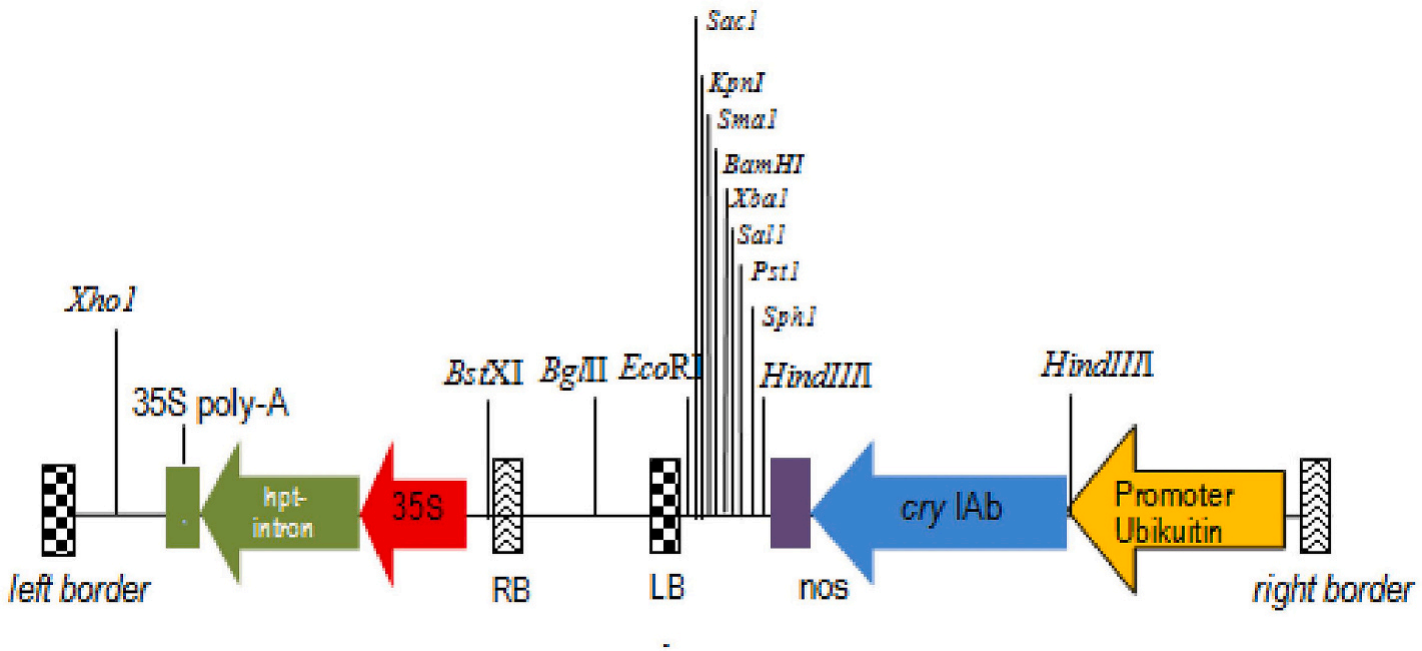
Structure of the pARBIO construct.

The study was conducted at the Genomics and Crop Improvement Laboratory of the National Research and Innovation Agency (BRIN), Indonesia. The binary vector pCAMBIA1300 served as the backbone for constructing a dual T-DNA expression system,^18^ as illustrated in Fig. 1. The primary T-DNA region contained the *cry1Ab* gene, which was placed under the control of *ubiquitin* promoter and *nos* terminator. The secondary T-DNA region incorporated the hygromycin phosphotransferase (*hpt*II) selectable marker gene driven by the CaMV *35S* promoter.

The construct of a double T-DNA vector involves inserting oligonucleotide adapters containing the Right Border (RB) and Left Border (LB) sequences into specific restriction sites (*Bst*XI and *Eco*RI) of the pCAMBIA1300 binary vector. This creates two independent T-DNA regions within a single plasmid, enabling Agrobacterium to transfer both segments into the plant genome; UBI: ubiquitin promoter; nos: nopaline synthase terminator.

A critical component of the vector design involved the synthesis of a specialized adapter containing complementary left border (LB) and right border (RB) sequences. The adapter oligonucleotides (Strand 1:5′-TGGGTAAACCTA AGAGAAAAGAGCGTTTAAGATCTTGGCAGGATATATTGTGGTGTAAACAG-3′; Strand 2: 5′-CATGCTGTTTACACCACAATATATCCTGCAAGATCTT AAACGCTCTTTTCTCTTAGGTTACACCAG-3′) were designed to facilitate precise assembly of the dual T-DNA system. The adapter was first introduced into the *BstXI* and *EcoRI* restriction sites of the pCAMBIA1300 backbone, creating an intermediate vector designated pARBIO. Subsequently, the complete *cry1Ab* expression cassette was inserted to generate the final transformation vector, pARBIO (Fig. 1). For genetic transformation, the pARBIO vector was introduced into *Agrobacterium tumefaciens* strain Agl-1 via tri-parental mating using *Escherichia coli* DH5α harboring the helper plasmid pRK2013.

### 2.2 Callus transformation of Rojolele cultivar

For callus induction, dehusked seeds of the aromatic *javanica* rice cultivar *Rojolele* were surface-sterilized through sequential treatment with 70% ethanol for 1 min, followed by a sterile distilled water rinse, and then 2% sodium hypochlorite for 30 min, with six subsequent 15-min sterile water rinses. The sterilized seeds were aseptically transferred to Petri dishes containing IK3 medium (LS medium supplemented with 2.5 mg/L 2,4-D) and incubated at 28 °C in complete darkness. After 7 days, the developing scutella were carefully excised and transferred to fresh IK3 medium. Following 14 days of culture under the same conditions, embryogenic calli were selected and subcultured for an additional 4 days to obtain optimal transformation-competent callus tissue.

For *Agrobacterium*-mediated transformation, *A. tumefaciens* strain Agl-1 harboring the pARBIO plasmid was cultured on AB medium plates for 3 days at 28 °C. Bacterial cells were then harvested and resuspended in AAM liquid medium supplemented with 100 μM acetosyringone, adjusting the suspension to an OD600 of 1.0. Approximately 300 embryogenic calli were immersed in the bacterial suspension for 15 min, after which excess liquid was removed by blotting on sterile filter paper. The calli were then co-cultivated on IK3 medium containing 100 μM acetosyringone for 3 days at 28 °C in the dark, following the protocol established by.^19^

Following co-cultivation, calli were transferred to primary selection medium I (IK3 + 250 mg/L cefotaxime +50 mg/L hygromycin) and maintained at 28 °C in complete darkness for 14 days. Surviving calli were then subcultured onto secondary selection medium (IK3 + 50 mg/L cefotaxime +50 mg/L hygromycin) under identical conditions for an additional 14 days. Resistant calli were subsequently transferred to regeneration medium (R3B: modified IK3 medium supplemented with 0.5 mg/L indole-3-acetic acid [IAA] and 0.3 mg/L 6-benzylaminopurine [BAP]), with biweekly subculturing under the same environmental conditions.

Developing plantlets were carefully transferred to Murashige and Skoog (MS) basal medium and incubated under continuous light (28 °C) to promote root development. Once established, plants were acclimatized by gradual exposure to ambient conditions before being transplanted into a sterile soil mixture in pots. All plants were subsequently maintained in a controlled greenhouse environment for further growth and analysis.

### 2.3 Transgenic screening

#### 2.3.1 PCR analysis

Genomic DNA was isolated from transgenic rice generations T_0_ to T_3_ using a modified CTAB method.^20^ PCR amplification confirmed the integration of *cryIAb* and *hpt* genes, using specific primers: for *cry1Ab* (forward: 5′-CATTGTGTCTCTCTTCCC-3′; reverse: 5′-CCGTTAGAGAAGTTGAAAGG-3′) and for *hpt* (forward: 5′-GATGCCTCCGCTCGAAGTAGCG-3′; reverse: 5′-GCATCTCCCGCCGTGCAC-3′). The PCR process was performed using the Dream Taq Green Master Mix (Thermo Fisher Scientific). Each 15 μL PCR reaction contained: 1× PCR buffer (with Mg^2+^), 0.05 mM dNTPs, 0.05 U μL^−1^ Taq DNA polymerase, 2.5 ng μL^−1^ each of forward and reverse primers, 1 μL template DNA, and nuclease-free water to adjust the final volume.

The thermal cycling protocol consisted of: initial denaturation at 95 °C for 5 min; 40 cycles of denaturation (95 °C, 1 min), annealing (55 °C, 1 min), and extension (72 °C, 1 min); followed by a final extension at 72 °C for 10 min. Expected amplicon sizes were 1012 bp for *cryIAb* and 492 bp for *hpt*. PCR products were separated by electrophoresis on 0.8% agarose gels in 0.5× TBE buffer (54 g Tris base, 27.5 g boric acid, and 20 mL 0.5 M EDTA [pH 8.0] per liter of 5× stock solution). Electrophoresis was conducted at 100 V for 1 h, and DNA bands were visualized using a Bio-Rad Gel Doc UV 1000 documentation system.

For segregation analysis, individual T_2_ progeny plants were screened by PCR using the same primer sets to determine the inheritance pattern of the *cry1Ab* and *hpt* transgenes. The presence or absence of each gene was recorded for each progeny plant, and the segregation ratios were calculated accordingly.

#### 2.3.2 Southern blot analysis

To confirm stable integration of the *cryIAb* transgene in T_3_ generation transgenic rice, Southern hybridization was performed using the AlkPhos Direct Labeling and Detection System (GE Healthcare) with CDP-Star chemiluminescent substrate. Genomic DNA (20 μg) from T_3_ generation transgenic plants was digested overnight with appropriate restriction enzymes *Hin*dIII, which has a recognition site within the *cry1Ab* gene cassette or the T-DNA region of the pARBIO construct. Therefore, this analysis confirms stable integration but does not allow determination of transgene copy number.

The digested DNA fragments were separated by 0.8% agarose gel electrophoresis and subsequently transferred to a nylon membrane via capillary blotting. The *cry*IAb-specific probe was labeled using the AlkPhos direct labeling system according to the manufacturer's protocol. Hybridization was carried out at 55 °C overnight, followed by stringent washes to remove non-specific binding.

Detection was performed using the CDP-Star chemiluminescent substrate, and signals were captured on X-ray film. The analysis confirmed stable integration of the *cry1Ab* transgene in selected T_3_ transgenic lines, as evidenced by discrete hybridization bands.

#### 2.3.3 Immunostrip assay

The expression of Cry1Ab protein in transgenic rice plants was verified at third generation using a commercial Bt Cry1Ab immunostrip test kit (Agdia USA) according to the manufacturer's protocol.^21^ Leaf tissues were collected from PCR-positive transgenic plants that showed successful integration of the *cry1Ab* gene. The immunostrip assay was performed to confirm the presence of the expressed Cry1Ab protein in these transgenic lines.

#### 2.3.4 Bioassay for yellow stem borer resistance evaluation

The bioassay was conducted under biosafety containment conditions to evaluate yellow stem borer resistance in a transgenic rice line at T_3_ generation. Plants were cultivated in potted soil media, with each pot enclosed in a plastic cylinder to prevent cross-contamination between the larval population. Larvae of the yellow stem borer (*S. incertulas*) were obtained from egg masses collected in rice fields and subsequently hatched under controlled laboratory conditions. At six weeks after planting (WAP), both transgenic *Rojolele* lines and wild-type control plants were artificially infested with five first-instar larvae per tiller, using three tillers per line to ensure consistent infestation pressure. The intensity of pest attacks was observed at 2, 3, and 4 weeks after inoculation (WAI), and plant resistance was assessed using the formula in accordance with the Standard Evaluation System.^22^

Damage severity was quantified by calculating the D value:

D = % Damage plants of lines tested x 100%.

% Damage plants of control susceptible plants.

Damage ratings were classified on a 0–9 scale: 0 = no damage, 1 = 1–20% damage (highly resistant), 3 = 21–40% damage (moderately resistant), 5 = 41–60% age (slightly resistant), 7 = 61–80% damage (susceptible), 9 = 81–100% and above damage (highly susceptible). Lines with D values ≤5 were considered resistant, while those ≥7 were classified as susceptible. Representative plants from each line were dissected at every treatment interval (2, 3, and 4 weeks after infestation) to assess larval development and feeding damage patterns.

### 2.4 Agronomic trait evaluation of transgenic Rojolele rice

The agronomic performance of transgenic *Rojolele* lines was assessed under controlled conditions alongside wild-type (WT) controls. Each line, including the WT, was planted with five biological replicates (*n* = 5 per line). Key growth parameters: plant height, number of productive tillers, flag leaf dimensions, panicle length, grains per panicle, and thousand-grain weight were recorded at critical developmental stages to evaluate overall growth and yield performance. Plant height was recorded from the soil surface to the panicle tip at maturity, while productive tillers were counted during harvest. Flag leaf length and width were measured on the uppermost fully expanded leaf using digital calipers. For yield assessment, 1000-grain weight was determined from oven-dried seeds (14% moisture content). Data were analyzed using JASP 0.19.3.0 software, with one-way ANOVA followed by Tukey's HSD post hoc test (*p* < 0.05) to determine significant differences between transgenic and wild-type (WT) lines.

## 3 Results

### 3.1 Transformation Efficiency and Regeneration

**Fig. 2:**
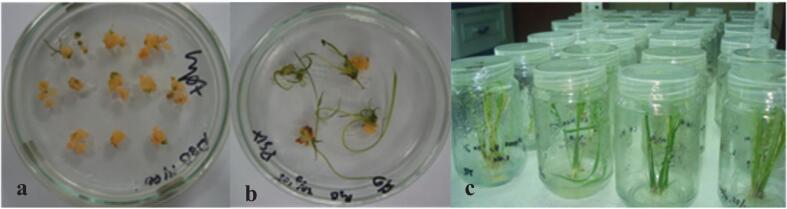
Key stages in the transformation of *Javanica* rice cv. Rojolele. (a) Embryogenic calli are induced from scutellum-derived tissues. (b) Shoot regeneration from hygromycin-resistant calli (c) Healthy T_0_ generation plantlets before acclimatization in soil.

The co-transformation of Rojolele embryogenic calli with *Agrobacterium tumefaciens* strain Agl-1 carrying the dual T-DNA vector pARBIO resulted in a regeneration efficiency of 12.3%, compared to 16% for the control vector pCAMBIA1300 (Table 1). Callus cultures transferred to R3B medium successfully regenerated into plantlets, as shown in Fig. 2.

**Table 1:** Regeneration efficiency of *Javanica* rice cv. Rojolele following transformation with pARBIO and pCAMBIA 1300 vectors.

Plasmid	Inoculated calli	Regenerated calli	Regeneration (%)
pARBIO	300	37	12.3%
pCAMBIA 1300	300	48	16%

### 3.2 Molecular Characterization: PCR, Southern Blot, and Gene Expression

**Fig. 3:**
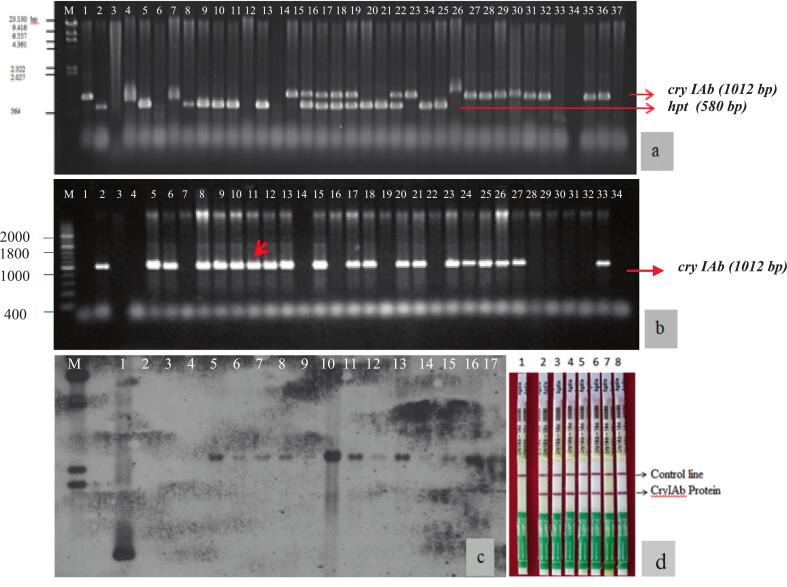
Molecular characterization of transgenic rice cv. Rojolele expressing *cry1Ab*.

PCR screening of T_0_ plants identified 21 independent transgenic events harboring both *cry1Ab* and *hpt* genes. From these, seven lines were selected for further analysis. In the T_1_ generation, the presence of *hpt* and *cry1Ab* genes was confirmed by PCR (Fig. 3a). Segregation analysis was continued in the T_2_ generation to further characterize the inheritance pattern of both transgenes (Fig. 3b). As shown in Table 2, among 210 T_2_ progeny plants analyzed, 84 (40.00%) carried only *cry1Ab* (marker-free), 56 (26.66%) carried only *hpt*, 47 (22.38%) carried both genes, and 23 (10.95%) were negative for both. The presence of plants carrying only *cry1Ab* in the T_2_ generation confirms the successful segregation of the two T-DNAs and the stability of the marker free phenotype. These results support the Mendelian inheritance of the transgenes and the effectiveness of the dual T-DNA vector strategy for producing marker-free transgenic rice lines.

**Table 2:** Segregation analysis of transgenic plants in the T_2_ generation.

lines	*cry* IAb + hpt	*cry*IAb	*hpt*	negative
I.AR.1	5	12	10	3
I.AR.2	7	13	5	5
I.AR.3	4	11	9	6
I.AR.4	9	14	5	2
III.AR.5.3	4	12	10	4
III.AR.5.2	10	12	8	0
IV.AR.3.1	8	10	9	3
	47 (22.38%)	84 (40.00%)	56 (26.66%)	23 (10.95%)

Southern blot analysis using *Hin*dIII was performed to assess transgene integration in the T_3_ generation. All transgenic lines showed hybridizing bands, confirming stable integration of the *cry1Ab* transgene (Fig. 3c). However, because *Hin*dIII has a recognition site within the *cry1Ab* cassette and the T-DNA region of the pARBIO construct, the resulting fragments are of similar size across different lines. Therefore, these data confirm stable integration but do not allow determination of transgene copy number. Immuno-strip assays validated the expression of *Cry1Ab* protein in T_3_ plants (Fig. 3d).

(a) PCR screening of T_1_ generation plants using *cry1Ab*- and *hpt*-specific primers. Lanes: M, λ/*Hin*dIII marker; 1, *cry1Ab* positive control; 2, *hpt* positive control; 3, negative control; 4–37 transgenic lines.
(b) PCR confirmation of *cry1Ab* integration in the T_2_ generation. Lanes: M, DNA marker; 1, negative control; 2, *cry1Ab* positive control; 3, water control; 4, non-transgenic control; 5–34 transgenic plant samples.
(c) Southern blot analysis of T_3_ generation transgenic rice plants using a *cry1Ab*-specific probe. Genomic DNA was digested with *Hin*dIII. Lanes: M, λ/HindIII size marker; 1, *cry1Ab* fragment control; 2, plant negative control; 3, non-transgenic plant; 4–5 (I.AR.1); 6–7 (I.AR.2); 8–9 (I.AR.3); 10–11 (I.AR.4); 12–13 (III.AR.5.2); 14–15 (III.AR.5.3); 16–7 (IV.AR.3.1) transgenic plant. The positive control did not produce a detectable band, most likely due to insufficient DNA quantity or partial degradation. However, all transgenic lines showed clear hybridizing bands, while the non-transgenic control showed no signal, confirming stable integration of the *cry1Ab* transgene.
(d) Immunostrip detection of Cry1Ab protein of T_3_ transgenic lines. Lanes: 1, non-transgenic control; 2–8, transgenic lines (I.AR.1, I.AR.2, I.AR.3, I.AR.4, III.AR.5.2, III.AR.5.3, IV.AR.3.1).

### 3.3 Bioassay and resistance efficacy

**Fig. 4:**
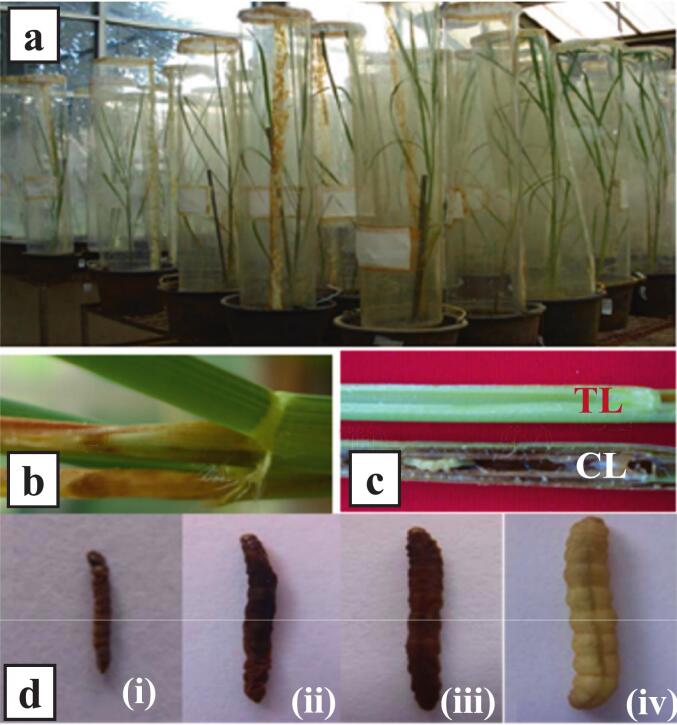
Bioassay evaluation of transgenic rice cv. Rojolele resistance against yellow stem borer (*Scirpophaga incertulas*) under contained conditions. (For interpretation of the references to colour in this figure legend, the reader is referred to the web version of this article.)

Bioassays against *S. incertulas* larvae revealed significant variability in resistance among the seven marker-free *cry1Ab* lines (Table 3; Fig. 4). Lines I.AR.4 and III.AR.5.3 exhibited the highest resistance levels, while I.AR.2 and III.AR.5.2 remained susceptible. Line IV.AR.3.1 showed intermediate resistance.

**Table 3:** Resistance classification of *cry1Ab*-expressing rice cv. Rojolele against the yellow stem borer at the vegetative stage.

Lines	Week observation						General categories
	Resistance index value (D)			Scoring			
	2	3	4	2	3	4	
I.AR-1	8.1	22.2	19.7	1	5	3	Moderate
I.AR-2	81.2	40.0	35.2	9	7	7	Susceptible
I.AR-3	50.0	13.4	11.9	7	3	3	Moderate
I.AR-4	0	0	0	0	0	0	Resistant
III.AR.5.2	92.8	35.2	39.5	9	7	7	Susceptible
III.AR.5.3	0	0	0	0	0	0	Resistant
IVAR.3.1	0	0	7.6	0	0	1	Resistant
Rojolele (wt)	26,3	36,8	53,3	9	7	8	Susceptible

(a) Experimental setup showing plants isolated in plastic cylinders to prevent cross-contamination; (b) Infestation protocol with two larvae per tiller at 61 days post-transplantation; (c) Comparative damage assessment between transgenic (TL) and non-transgenic control (CL) lines. (d) The symptoms of *S. incertulas* larvae feeding on: (i-iii) transgenic rice plants, (iv) non-transgenic rice plants. Red scale bar = 5 mm.

### 3.4 Agronomic performance

Evaluations of key agronomic traits demonstrated that *cry1Ab* expression did not adversely affect plant morphology or yield components compared to wild type (controls). The analysis revealed no significant differences were observed between transgenic lines and the wild type for all agronomic traits assessed (Table 4).

**Table 4:** The agronomic performance of transgenic lines.

Lines	Plant height (cm)	Productive tillers	Flag - length (cm)	Flag width (cm)	Panicle length (cm)	Grain/ panicle	1000-GW (gr)
I.AR.1	144.40^a^	7.40^a^	38.54^ab^	1.68^a^	28.10^bc^	100.00^a^	29.28^a^
I.AR.2	143.00^a^	7.80^a^	39.96^b^	1.68^a^	24.86^abc^	68.40^a^	28.50^a^
I.AR.3	139.00^a^	8.20^a^	38.78^ab^	1.70^a^	27.96^bc^	91.60^a^	29.63^a^
I.AR.4	138.00^a^	8.20^a^	33.52^a^	4.70^a^	22.06^a^	78.20^a^	29.14^a^
III.AR.5.2	140.40^a^	8.00^a^	36.30^ab^	1.56^a^	22.42^a^	68.00^a^	28.60^a^
III.A.R.5.3	142.60^a^	7.40^a^	41.00^b^	1.66^a^	29.50^c^	103.20^a^	28.60^a^
IV.AR.3.1	142.00^a^	8.20^a^	40.42^b^	1.58^a^	25.62^abc^	86.80^a^	28.10^a^
Rojolele	140.40^a^	8.20^a^	36.68^ab^	1.62^a^	23.42^ab^	64.40^a^	29.56^a^

Note: Different lowercase letters indicate significant differences (p < 0.05) between lines (Tukey's test).

## 4 Discussion

The observed regeneration efficiency of 12.3% following co-transformation of Rojolele embryogenic calli with *Agrobacterium tumefaciens* (Agl-1) carrying the dual T-DNA vector pARBIO was lower than the 16% efficiency obtained with the control vector pCAMBIA 1300 (Table 1; Fig. 2). This result reflects the well-documented genotype-dependent variability in transformation responses, particularly in javanica rice, where hormonal balance and callus quality are pivotal factors influencing regeneration outcomes^23^˒.^24^ The observed co-transformation efficiency is consistent with the 38.5% rate reported for the generation of marker-free transgenic indica rice using a two T-DNA system, highlighting the potential of this strategy despite inherent efficiency trade-offs.^11^ The slight reduction in efficiency observed with pARBIO may be attributed to the metabolic burden imposed by co-transforming two T-DNAs, a phenomenon previously reported in complex plasmid systems such as CRISPR/Cas9-based transformations, where increased plasmid complexity can negatively impact cell viability and regeneration.^25^ The effective promotion of embryogenic callus proliferation by the R3B medium corroborates earlier findings that compact, nodular calli exhibit higher regenerative potential.^26^ Moreover, the auxin-cytokinin cross-talk maintained at an IAA:BAP ratio of 1:0.6 aligns with optimized somatic embryogenesis protocols across various crops, underscoring the universal role of hormonal regulation in regeneration.^27^ These combined factors emphasize the need for genotype-specific media optimization to enhance transformation rates in Rojolele rice.^28^

Molecular characterization confirmed the generation of stable transgenic Rojolele lines expressing *cry1Ab*. PCR screening of T_0_ plants identified 21 independent transgenic events harboring both *cry1Ab* and *hpt* genes. From these, seven lines were selected at the T_1_ generation for further characterization based on their robust growth and positive transgene detection.

To evaluate the inheritance pattern and segregation of the two T-DNA, progeny of these seven selected lines was subjected to PCR analysis in the T_2_ generation. As shown in Table 2, segregation analysis of T2 progeny revealed that 40% of the plants were marker-free (carrying only *cry1Ab*). The presence of plants carrying only *cry1Ab* in the T2 generation confirms the successful separation of the two T-DNAs and the stability of the marker-free phenotype.

The observed segregation pattern follows Mendelian inheritance, indicating that the two T-DNAs integrated into unlinked loci and segregated independently during gamete formation. This result is consistent with the expected behavior of a dual T-DNA vector system. The marker-free efficiency of approximately 40% obtained in this study is comparable to other marker-excision technologies, such as the Cre/lox system and co-transformation approaches reported in pigeon pea and indica rice, respectively^29^˒.^11^

To further contextualize our findings, we compared our results with recent studies utilizing double T-DNA systems. A recent study demonstrated that spacer length between two T-DNAs critically influences linked integration frequency, with a ∼ 3 kb spacer eliminating linked integration (0%) compared to 35% with a 119 bp spacer.^30^ Although our vector design differed in spacer configuration, the successful segregation of T-DNAs in 40% of our T_2_ progeny aligns with the observation that tandem orientation and adequate spacer length promote independent integration. Furthermore, another study reported an impressive 55.6% homozygous marker excision in rice using a CRISPR/Cas9-mediated HDR system driven by a meristem-specific promoter, achieving 82.2% total excision efficiency in T1 progeny.^31^ While our system achieved 40% marker-free plants through Mendelian segregation without the need for additional genetic elements, the auto-excision approach offers the advantage of eliminating the need for progeny screening, albeit with more complex vector construction.

Southern blot analysis provides critical confirmation of stable transgene integration, a prerequisite for predictable gene expression. By ensuring that integration events are consistent, this approach minimizes the likelihood of transcriptional silencing and strengthens the reliability of transgene performance. Furthermore, Southern blot validation is an essential component of molecular characterization required for regulatory approval, thereby facilitating the advancement of transgenic lines toward field evaluation and commercialization.^9^˒.^32^ Finally, the functional expression of the integrated *cry1Ab* gene was verified at the protein level. Immuno-strip assays performed on leaf tissue from T_3_ plants confirmed the presence of the *Cry1Ab* toxin (Fig. 3d). This detection confirms that the transgene is transcriptionally active and that the resulting protein is accumulated to detectable levels.

The correlation between molecular confirmation, successful marker excision, and protein expression establishes a solid foundation for expecting functional insect resistance, a principle demonstrated in other Bt crops where protein detection directly correlates with bioassay efficacy^13^˒^33^˒.^34^ Similar confirmation of functional protein expression via ELISA was a key step in validating the efficacy of the *cry2AX1* marker-free lines developed.^11^

Bioassay evaluation of the seven marker-free *cry1Ab*-expressing Rojolele lines revealed significant variation in resistance against *Scirpophaga incertulas* larvae (Table 3, Fig. 4). Two lines, I.AR.4 and III.AR.5.3, demonstrated the highest level of resistance (Scale 0), with no visible symptoms of damage observed on the plants. In contrast, I.AR.2 and III.AR.5.2 remained susceptible (Scale 7–9), while IV.AR.3.1 showed moderate resistance. This spectrum of efficacy is a common feature in transgenic crop development and underscores the importance of event selection, as transgene expression can be markedly influenced by the genomic insertion site, a phenomenon known as the positional effect.^9^ The superior performance of the resistant lines aligns with the established mode of action of *Cry1Ab* protein, which, upon ingestion and activation in the alkaline insect midgut, binds to specific receptors (e.g., cadherin, aminopeptidase-N) leading to pore formation, osmotic imbalance, and eventual larval mortality^7^˒.^35^ The observed reduction in plant damage symptoms on resistant lines is likely direct consequences of this toxic pathway. The variability among our lines likely correlates with differences in *Cry1Ab* protein accumulation, which is a key determinant of Bt efficacy.^5^ Similar event-dependent variability in insect resistance, linked directly to Bt protein expression levels, has been documented in other systems, such as transgenic rice expressing *Cry*2AX1 against *Chaphalocrocis medinalis*^36^ and in the evaluation of marker-free *cry2AX1* lines, where protein concentration directly correlated with larval mortality against both the yellow stem borer and leaffolder.^11^

The clear resistance hierarchy (I.AR.4 & III.AR.5.3 > IV.AR.3.1) mirrors patterns observed in other successful Bt rice programs. For instance, elite commercial Bt rice lines like Huahui No.1 (expressing *Cry1Ab*/Ac) have shown high and consistent field resistance against major lepidopteran pests, including *S. incertulas*, resulting in significant yield protection.^38^ This consistency is often associated with single-copy transgene integration events, which tend to provide more stable and predictable expression.^14^ It is suspected that the susceptibility of lines I.AR.2 and III.AR.5.2 may therefore be attributed to suboptimal transgene expression, potential silencing mechanisms, or low protein accumulation. This underscores the necessity for comprehensive molecular and phenotypic screening to identify superior, field-ready transformation events.^13^ Although controlled bioassays offer strong preliminary evidence, they may not accurately predict performance under field conditions. The durability and stability of insect resistance must therefore be validated through multi-location field trials that expose plants to natural pest pressures and environmental variability, a standard and critical phase in transgenic crop development.

A critical assessment of agronomic performance confirmed that the integration and expression of the *cry1Ab* transgene did not compromise the essential morphological and yield-related traits of the Rojolele cultivar. Evaluation of key parameters, including plant height, number of productive tillers, flag leaf dimensions, panicle length, grains per panicle, and 1000-grain weight, revealed that all seven transgenic lines were largely comparable to the non-transgenic wild-type control (Table 4). No consistent negative effect of the transgene was observed across the evaluated lines. Notably, some lines, such as IV.AR.3.1, performed equivalently or even exhibited slight, nonsignificant enhancements in certain traits compared to the control, underscoring the absence of detrimental pleiotropic effects or yield penalties. This preservation of agronomic integrity aligns with the established principle of substantial equivalence for genetically modified crops. Comprehensive studies on commercial and pre-commercial Bt rice lines have consistently demonstrated that constitutive or tissue-specific expression of *Cry* proteins, when properly regulated, does not adversely affect key growth, yield, or quality metrics.^37^ A recent example is the marker-free *cry2AX1* transgenic rice, which showed no significant differences in plant height, tiller number, leaf count, or single plant yield compared to its non-transgenic.^11^ Similar outcomes have been documented in other crops; for instance, transgenic soybean expressing a modified *cry1C* gene retained the parental phenotype, seed yield, and nutritional quality (protein and oil content), proving that targeted insect resistance can be engineered without an agronomic trade-off.^32^ Our findings in the aromatic *Japonica* background of Rojolele further extend this evidence, challenging the notion that genetic modification necessarily impairs the quality or productivity of specialty cultivars.^16^ Collectively, these results validate the feasibility of developing transgenic aromatic rice with robust insect resistance while safeguarding its valued agronomic and morphological identity. To fully ascertain the environmental stability and commercial potential of these lines, further investigations are imperative. These should include multi-location field trials across representative agroecological zones to evaluate performance under diverse stresses, alongside detailed biochemical profiling to confirm the retention of the volatile compounds responsible for Rojolele's premium aromatic quality.

The successful development of marker-free *cry1Ab* Rojolele lines represents a significant step toward addressing primary biosafety and regulatory concerns associated with transgenic crops. The absence of selectable marker genes (SMGs), particularly those conferring antibiotic resistance, mitigates a major hurdle for environmental risk assessment, public acceptance, and eventual commercialization^38^˒.^39^ Our application of a dual T-DNA strategy aligns with the global advancement of “clean gene” technologies, which are increasingly recognized as essential for the sustainable deployment of genetic engineering in agriculture. To ensure the long-term durability of resistance and preempt the evolution of pest populations, proactive management strategies must be implemented. Gene pyramiding stacking *cry1Ab* with other Bt genes possessing distinct modes of action, such as *cry1C*, *cry2A*, or *vip3A* is a proven strategy to broaden the spectrum of resistance and delay the selection of resistant insect biotypes^37^˒.^40^ An even more sophisticated approach involves stacking a Bt gene with an RNA interference (RNAi) mechanism. For instance, transgenic rice co-expressing *Cry*1Ca and dsRNA targeting the *p38* gene of *Chilo suppressalis* resulted in dramatically higher larval mortality than *Cry*1Ca expression alone, illustrating the efficacy of multi-mechanism pest control.^41^ Targeting essential insect genes like *CsV-ATPase* via RNAi presents a complementary and potent strategy for future trait enhancement.^42^ Prior to any commercial release, comprehensive field validation through multi-location trials is imperative. Such trials are crucial to evaluate the stability of yield protection under natural and variable infestations, as pest pressure and damage are highly influenced by crop phenology and local agroecosystems.^43^ Concurrently, rigorous ecological risk assessment must be conducted. This includes monitoring the impact on non-target arthropod communities, particularly beneficial predators and parasitoids. Reassuringly, existing studies on Bt rice and maize expressing novel *Cry* proteins (e.g., *Cry1Ab*1Gc) have reported no significant adverse effects on the diversity or population dynamics of non-target arthropods^34^˒.^37^ Collectively, these steps from strategic trait deployment to thorough environmental monitoring will be essential for the responsible and sustainable integration of these insect-resistant aromatic rice lines into Indonesian cropping systems.

The significance of our work is further highlighted when compared to other marker-free strategies in cereal crops. Co-transformation followed by doubled haploid technology in barley achieved 1.5 marker-free lines per 100 embryos using two separate plasmids in a single *Agrobacterium* clone.^44^ Our study achieved a marker-free efficiency of 40% in the T_2_ generation, demonstrating that the double T-DNA vector approach in Rojolele rice is highly competitive in terms of efficiency. The retention of agronomic traits in our marker-free lines, as confirmed by phenotypic analysis (Table 4), aligns with the principle that well-designed marker-free systems should not compromise plant performancer.^45^ Collectively, these comparisons position our study as a successful application of marker-free technology in a high value aromatic rice cultivar, bridging the gap between biosafety compliance and consumer preferred quality traits.

## 5 Conclusion

This study successfully developed marker-free, Cry1Ab-expressing Rojolele rice lines with complete resistance to *S. incertulas*. Key achievements include: Precision Engineering: Southern blot analysis confirmed that the transgenic plants were stably integrated into the plant genome. Integration of *cry1Ab* via the dual T-DNA vector strategy also enabled the elimination of the *hpt* selectable marker, resulting in marker-free transgenic lines. Biosafety Compliance: Alignment with global standards for transgenic crops. These results provide a sustainable model for protecting aromatic rice, bridging pest resistance and consumer demand. Future adoption will require field validation and stakeholder engagement to address regulatory and public acceptance barriers.

## CRediT authorship contribution statement

**Agus Rachmat:** Writing – review & editing, Writing – original draft, Supervision, Methodology, Funding acquisition, Data curation, Conceptualization. **Enny Rimita Sembiring:** Methodology, Investigation, Data curation. **Ade Nena Nurhasanah:** Methodology, Data curation. **Nurul Fitriah:** Methodology, Data curation. **Vincentia Esti Windiastri:** Methodology, Data curation. **Eva Erdayani:** Validation, Methodology, Data curation. **Rumella Simarmata:** Writing – review & editing, Validation, Formal analysis. **Inez H.S. Loedin:** Validation, Supervision, Conceptualization. **Sri Indrayani:** Methodology, Data curation. **Carla Frieda Pantouw:** Methodology, Data curation. **Siti Halimah Larengkeng:** Methodology, Formal analysis. **Enung Sri Mulyaningsih:** Writing – review & editing, Writing – original draft, Supervision, Methodology, Investigation.

## Funding

This research was funded by the Indonesia Endowment Fund for Education (LPDP) through the RIIM Batch 4 program, managed by the National Research and Innovation Agency (BRIN), Republic of Indonesia, under Grant number: 37/II.7/HK2023.

## Declaration of competing interest

The authors declare the following financial interests/personal relationships which may be considered as potential competing interests: Agus Rachmat reports financial support was provided by Indonesia Endowment Fund for Education (LPDP) through the RIIM (Riset dan Inovasi untuk Indonesia Maju) Batch 4 program. The other authors, including Enung Sri Mulyaningsih, declare that they have no known competing financial interests or personal relationships that could have appeared to influence the work reported in this paper.

## References

[1] Sanyal S., Subba Rao V.M., Timmanna H. (2025). The global invasion risk of rice yellow stem borer *Scirpophaga incertulas* Walker (Lepidoptera: Crambidae) under current and future climate scenarios. PLoS One.

[2] Zhou S., Luo G., Yang Q. (2024). A chromosome-level genome assembly of yellow stem borer (*Scirpophaga incertulas*). Sci. Data.

[3] Hamsein N.N., Yeo F.K.S., Sallehuddin R. (2020). Oviposition behaviour of *Scirpophaga incertulas* (Walker) (Lepidoptera: Pyralidae) on Sarawak rice landraces. Taiwania.

[4] Sánchez-Yáñez J.M., Rico J.L., Ulíbarri G. (2022). *Bacillus thuringiensis* (Bt) is more than a special agent for biological control of pests. J Appl Biotechnol Bioeng.

[5] Liu L., Boyd S.D., Bulla L.A., Winkler D.D. (2018). The defined toxin-binding region of the cadherin G-protein coupled receptor, BT-R1, for the active Cry1Ab toxin of *bacillus thuringiensis*. J Proteomics Bioinform.

[6] Ibrahim M.A., Griko N., Junker M., Bulla L.A. (2010). *Bacillus thuringiensis*: a genomics and proteomics perspective. Bioeng Bugs.

[7] Bravo A., Gill S.S., Soberón M. (2007). Mode of action of *bacillus thuringiensis* cry and Cyt toxins and their potential for insect control. Toxicon.

[8] Van Rie J., Jansens S., Jeschke P., Witschel M., Krämer W., Schirmer U. (2019). Modern Crop Protection Compounds.

[9] Hu Y., Tian C., Feng Y. (2024). Transgenic early japonica rice: integration and expression characterization of stem borer resistance Bt gene. Gene.

[10] Gebre W., Belachew B. (2021). Genetic engineering of the cry gene as a source of resistance to insect pest of some major crops. J Biol Agric Healthc.

[11] Rajadurai G., Kalaivani A., Sudhakar D., Udayasuriyan V., Natarajan N. (2025). Marker-free transgenic Bt rice engineered for resistance to yellow stem borer and leaffolder. Cereal Res. Commun..

[12] Tabashnik B.E., Brévault T., Carrière Y. (2013). Insect resistance to Bt crops: lessons from the first billion acres. Nat. Biotechnol..

[13] Chen H., Huang Y., Ye M., Wang Y., He X., Tu J. (2023). Achieving high expression of cry in green tissues and negligible expression in endosperm simultaneously via rbcS gene fusion strategy in rice. Int. J. Mol. Sci..

[14] Xu C., Cheng J., Lin H., Lin C., Gao J., Shen Z. (2018). Characterization of transgenic rice expressing fusion protein Cry1Ab/Vip3A for insect resistance. Sci. Rep..

[15] Kumar M., Prasad R. (2020).

[16] Dastan S., Ghareyazie B., Pishgar S.H. (2019). Environmental impacts of transgenic Bt rice and non-Bt rice cultivars in northern Iran. Biocatal Agric Biotechnol.

[17] Dwiningsih Y., Al-Kahtani J. (2022). Rojolele: a premium aromatic rice variety in Indonesia. Int J Agric Sci Technol.

[18] Roberts C.S., Rajagopal S., Smith L.A. (1998).

[19] Hiei Y., Ohta S., Komari T., Kumashiro T. (1994). Efficient transformation of rice (*Oryza sativa* L.) mediated by *Agrobacterium* and sequence analysis of the boundaries of the T-DNA. Plant J..

[20] van Heusden A.W., van Ooijen J.W., Vrielink-van Ginkel R., Verbeek W.H.J., Wietsma W.A., Kik C. (2000). A genetic map of an interspecific cross in *Allium* based on amplified fragment length polymorphism (AFLP) markers. Theor. Appl. Genet..

[21] Agdia Inc. (2003).

[22] International Rice Research Institute (2002).

[23] Ali J., Nicolas K.L.C., Akther S. (2021). Improved anther culture media for enhanced callus formation and plant regeneration in rice (*Oryza sativa* L.). Plants.

[24] Shimizu-Sato S., Tsuda K., Nosaka-Takahashi M. (2020). Agrobacterium-mediated genetic transformation of wild *Oryza* species using immature embryos. Rice.

[25] Liang Y., Biswas S., Kim B., Bailey-Serres J., Septiningsih E.M. (2021). Improved transformation and regeneration of Indica rice: disruption of SUB1A as a test case via CRISPR-Cas9. Int. J. Mol. Sci..

[26] Noor W., Lone R., Kamili A.N., Husaini A.M. (2022). Callus induction and regeneration in high-altitude Himalayan rice genotype SR4 via seed explant. Biotechnol Rep.

[27] Avilez-Montalvo J.R., Quintana-Escobar A.O., Méndez-Hernández H.A. (2022). Auxin–cytokinin crosstalk in somatic embryogenesis of *Coffea canephora*. Plants.

[28] Sidik N.J., Agha H.M., Alkamil A.A., Alsayadi M.M.S., Mohammed A.A. (2024). A mini review of plant tissue culture: the role of media optimization, growth regulators in modern agriculture, callus induction and the applications. AUIQ Complement Biol Syst.

[29] Ganguly S., Purohit A., Ghosh S., Chaudhuri R.K., Das S., Chakraborti D. (2022). Clean gene technology to develop selectable marker-free pod borer-resistant transgenic pigeon pea events involving the constitutive expression of *Cry1Ac*. Appl. Microbiol. Biotechnol..

[30] Al Nuaimi M., Rafi M., ElSiddig M., Aldarmaki M., George S., Amiri K.M.A. (2025). T-DNA orientation, distance between two T-DNAs, and the transformation target cells significantly impact vector backbone integration and efficiency of generating marker-free transgenic plants in a co-transformation system. Plant J..

[31] Tan J., Wang Y., Chen S. (2022). An efficient marker gene excision strategy based on CRISPR/Cas9-mediated homology-directed repair in rice. Int. J. Mol. Sci..

[32] Fang Q., Cao Y., Oo T.H. (2024). Overexpression of *cry1c* enhances resistance against soybean pod borer (*Leguminivora glycinivorella*) in soybean. Plants.

[33] Boddupally D., Tamirisa S., Gundra S.R., Vudem D.R., Khareedu V.R. (2018). Expression of hybrid fusion protein (Cry1Ac::ASAL) in transgenic rice plants imparts resistance against multiple insect pests. Sci. Rep..

[34] Liu Y., Han S., Yang S. (2022). Engineered chimeric insecticidal crystalline protein improves resistance to lepidopteran insects in rice (*Oryza sativa* L.) and maize (*Zea mays* L.). Sci. Rep..

[35] Xiao Y., Wu K. (2019). Recent progress on the interaction between insects and *bacillus thuringiensis* crops. Philos. Trans. R. Soc. Lond. Ser. B Biol. Sci..

[36] Rajadurai G., Sudhakar D., Varanavasiappan S., Balakrishnan N., Udayasuriyan V., Natarajan N. (2023). Adult oviposition preference and larval performance of *Cnaphalocrocis medinalis* Guenée (Pyralidae: Lepidoptera) on transgenic Bt rice. Int. J. Trop. Insect Sci..

[37] Li C., Wang J., Ling F., You A. (2023). Application and development of Bt insect resistance genes in rice breeding. Sustainability.

[38] Singh R., Kaur N., Praba U.P. (2022). A prospective review on selectable marker-free genome engineered rice: past, present and future scientific realm. Front. Genet..

[39] Li X., Pan L., Bi D. (2021). Generation of marker-free transgenic rice resistant to rice blast disease using ac/ds transposon-mediated transgene reintegration system. Front. Plant Sci..

[40] Kamatham S., Munagapati S., Manikanta K.N. (2021). Recent advances in engineering crop plants for resistance to insect pests. Egypt J Biol Pest Control.

[41] Wu Y., Weng Z., Yan H. (2023). The microRNA73225p/p38/Hsp19 axis modulates *Chilo suppressalis* cell defences against Cry1Ca: an effective target for a stacked transgenic rice approach. Plant Biotechnol. J..

[42] Qiu L., Wang P., Wu T. (2017). Downregulation of *Chilo suppressalis* alkaline phosphatase genes associated with resistance to three transgenic *bacillus thuringiensis* rice lines. Insect Mol. Biol..

[43] Dessky E.S., Ismail R.M., Elarabi N.I., Abdelhadi A.A., Abdallah N.A. (2021). Improvement of sugarcane for borer resistance using Agrobacterium-mediated transformation of *cry1Ac* gene. GM Crops Food.

[44] Kapusi E., Hensel G., Coronado M.-J. (2013). The elimination of a selectable marker gene in the doubled haploid progeny of co-transformed barley plants. Plant Mol. Biol..

[45] Talukder A.H., Mahmud S., Shibly A.Z. (2015). Production of the marker free transgenic plants – an update review. Journal of Pharmacognosy and Phytochemistry.

